# Fluoride Release and Rechargeability of Poly(lactic acid) Composites with Glass Ionomer Cement

**DOI:** 10.3390/polym15204041

**Published:** 2023-10-10

**Authors:** Sudarat Wongphattarakul, Rungroj Kuson, Thanapat Sastraruji, Kullapop Suttiat

**Affiliations:** 1 Department of Prosthodontics, Faculty of Dentistry, Chiang Mai University, Chiang Mai 50200, Thailand; sudarat_wong@cmu.ac.th (S.W.); rungroj_kuson@cmu.ac.th (R.K.); 2 Dental Research Center, Faculty of Dentistry, Chiang Mai University, Chiang Mai 50200, Thailand; thanapat.s@cmu.ac.th

**Keywords:** poly(lactic acid), glass ionomer cement, fluoride release, fluoride recharge, anticariogenic material

## Abstract

This study investigates the fluoride release, rechargeability and degradation behaviors of newly developed anticariogenic poly(lactic acid) (PLA) composites. The PLA composite with various concentrations (0%, 5%, 10%, 15% and 20% by weight) of glass ionomer cement (GIC) and sodium fluoride (NaF) were prepared using solvent casting method. The fluoride release, fluoride rechargeability and degradation behavior were evaluated. All experimental groups demonstrated fluoride-releasing ability. The highest level of fluoride ions released was found in PLA composite with sodium fluoride (PLA/NaF). Following the 28-day period, both groups showed a gradual reduction in fluoride ion released, ranging between 0.03 ± 0.01 and 0.53 ± 0.06 ppm, although remaining within the effective range for tooth remineralization. However, the rechargeability was only observed in PLA composite with GIC (PLA/GIC). Following an eight-week in vitro degradation test, all PLA/NaF groups displayed a significantly higher percentage of weight change and water absorption compared to the PLA/GIC and the control group. In SEM analysis, the formation of surface porosities was clearly noticed in all PLA/NaF. All specimens retained their structural integrity throughout the study. In conclusion, the newly developed PLA/GIC displays promising possibilities as an anticariogenic material. Furthermore, the rechargeability of these ions are repeatable, ensuring their long-term utility.

## 1. Introduction

Dental caries is one of the most prevalent chronic diseases, affecting more than 40% of the world’s population [1]. The correlation between aging and the increase in caries lesions has been extensively examined and reported in previous articles [2]. The common age-related conditions, including decreased salivary flow, the presence of chronic illnesses, the use of multiple medications and compromised physical control, contribute to an increase in dental caries susceptibility in older adults [3].

As is already known, the early onset of carious lesions can be effectively repaired through a remineralization process [4]. This repairing process involves the redeposition of calcium and phosphate ions from the surrounding environment into the gaps within the demineralized tooth structure. As a result, there is an increase in the overall mineral accumulation and apposition of hydroxyapatite crystals [5]. In the presence of fluoride ions, hydroxyl ions within hydroxyapatite are replaced by fluoride ions. Consequently, fluorapatite is formed, resulting in a reduction in mineral solubility and an enhancement in mechanical strength. It is widely accepted that this mechanism leads to the reduction in enamel demineralization and promotes tooth remineralization [4,5].

The fluoridated hydroxyapatite, having a lower critical pH level, demonstrates increased resistance to the initiation of acid demineralization and the development of carious lesions [6]. In addition, the previous report also addressed the bacteriostatic effect of fluoride in solution [7]. Therefore, the fluoride ions provide both inhibiting effects on acid tooth demineralization and bacterial acid production. Moreover, fluoride ions accelerate the formation of new fluorapatite crystals, which aids in remineralization [8]. 

The best approach to caries control as suggested by Sjogren et al. is a sustained supply of low levels of fluoride for an extended period [9]. To achieve this desired property, fluoride is typically delivered through everyday dental products like mouthwashes, toothpastes and gels [10]. However, because of the rapid dissolution and clearance of fluoride agents from the oral cavity and tooth surfaces, multiple exposure to the fluoride therapy is necessary to maintain therapeutic levels of fluoride ions to prevent or treat dental caries [11]. Thus, the incorporation of fluoride into oral appliances that are directly in contact with the tooth surface is a solution to provide the long-term release of fluoride ions to enhance the remineralization of early carious lesions. 

To address these issues, several research have incorporated fluoride into poly(methyl methacrylate) (PMMA) to develop fluoride-releasing materials. Fluoride in various forms, such as sodium fluoride (NaF), calcium fluoride, amine fluoride, glass ionomer cement (GIC) or pre-reacted glass ionomer cement, can be added to PMMA [12,13,14,15]. Fluoride-releasing polymeric materials can serve as a fluoride reservoir, elevating fluoride levels in saliva, plaque and dental hard tissues [12]. According to previous studies, fluoride-releasing dentures can offer adequate fluoride ions to prevent dental caries by enhancing remineralization and inhibiting demineralization [16,17]. Agarwal et al. conducted a study to evaluate the fluoride release of the PMMA denture resin with NaF. The findings indicated that the inclusion of 20 wt% NaF resulted in a sufficient level of fluoride ions for tooth remineralization. Nevertheless, the researchers noted a significant decrease in the amount of fluoride released after a period of 3 days, with a reduction of approximately 50% compared to the first hour [16].

Thus, maintaining the ion-releasing effect for a long duration has still not been attainable [17]. The incorporation of GIC is an interesting choice for developing material with long fluoride-releasing ability. GICs are derived from weak poly(acrylic acid) and fluoroaluminosilicate (FAS) glass. When the powder and liquid are combined, an acid-base reaction occurs, resulting in the dissolution of the glass phase, thus resulting in the crosslinking of the poly(acrylic acid) chain to calcium ions, which are later replaced by aluminum ions. Notably, fluoride ions do not participate in the cross-linking in the cement and are dispersed in the matrix of the cement [18]. The amorphous structure of GIC allows for an exchange of ions with the surrounding liquid medium, thereby facilitating fluoride release [19]. Besides its fluoride release ability, the long-term sustainability of fluoride ion release and the potential for fluoride ion reabsorption are key advantages of GICs compared to other fluoride-providing particles [20]. These properties overcome the issue of fluoride depletion commonly observed in conventional fluoride-releasing materials. The consistent and prolonged release of fluoride ions from materials has been proved as the basic requirement for anticariogenic material [9].

As previously stated, fluoride can be added to PMMA, but poly(lactic acid) (PLA) is an emerging dental material. This semi-crystalline thermoplastic bio-sourced polymer has gained popularity because of its biocompatibility and biodegradability [21]. Moreover, studies have shown that PLA polymer offers acceptable mechanical properties in some dental applications and meets the requirement of the International Organization for Standardization (ISO) 20795-1 standards [22]. This polymer is frequently employed as a biomaterial for medical and dental applications [23]. In addition, its biocompatibility, mechanical properties and ability to tailor the degradation rate make it a compelling option for use in tissue engineering, drug delivery systems and implantable medical devices [23]. Studies have been incorporating different ingredients into the PLA to produce different blends of polymer. For instance, Jiang et al. fabricated a drug delivery system that helps treat attention-deficit hyperactivity disorder (ADHD) by introducing clonidine hydrochloride into PLA to fabricate drug-loaded orthodontic retainers for the sustained release of drugs [24]. 

However, studies have not incorporated fluoride into PLA polymer to prevent dental caries. Therefore, this research study aims to incorporate PLA with GIC (PLA/GIC) in various concentrations and investigate the fluoride release, rechargeability and degradation.

## 2. Materials and Methods

### 2.1. Materials 

The poly(lactic acid) (PLA 4043D) supplied by NatureWorks (Plymouth, MN, USA) was used as the matrix of composites. NaF was supplied by Merck (Rahway, NJ, USA) and GIC Fuji VII was supplied by GC Corporation (Tokyo, Japan). Total ionic strength adjustment buffer III was purchased from Thermo Orion (Beverly, MA, USA). Phosphate-buffered saline (PBS) was purchased from Sigma-Aldrich (St. Louis, MO, USA). Chloroform was purchased from Union Science (Chiang Mai, Thailand). All products were used without further purification.

### 2.2. Specimen Preparation

**Figure 1:**
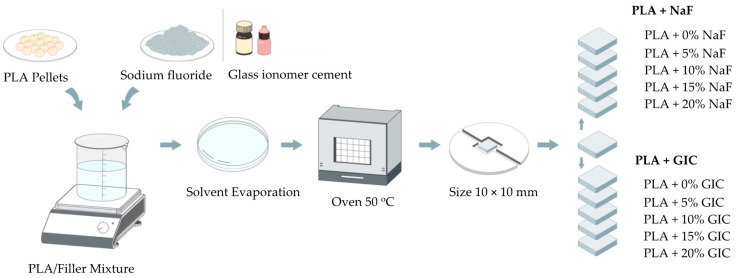
A schematic illustration of specimen preparation.

Four hundred films were prepared using the solvent casting method. Ten groups were studied by combining PLA with different filler materials (NaF and GIC), with varying filler content (5, 10, 15 and 20 wt%), as depicted in Figure 1. Pure PLA were used as control samples. 

For film preparation, PLA pellets were dissolved in chloroform (10 wt%) utilizing a magnetic stirrer at room temperature for 8 h until a complete homogenous and transparent solution was formed. Once the PLA solution was obtained, different filler materials (NaF and GIC) with different filler content (5, 10, 15 and 20 wt%) were introduced to the solution and mixed at room temperature for 1 h. Then, 15 mL of polymer–filler mixture was cast in a Petri dish and left for solvent evaporation in the fume hood at room temperature for at least 24 h. The polymer blend was then dried overnight in a vacuum oven at 50 °C to remove any residual solvent and moisture. After that, thin films were cut into small pieces of 10 mm × 10 mm and were further analyzed.

### 2.3. Specimen Characterization

#### 2.3.1. Fluoride Release

The 10 × 10 mm polymer films (*n* = 10) were placed in an individual plastic bottle with a cap containing 5.0 mL of deionized water for 28 days at 37 °C. The fluoride ion concentrations were measured at daily intervals for 4 weeks using Thermo scientific Orion in combination with an ion meter (Orion model 4-Star benchtop pH meter, Thermo Fisher Scientific, Waltham, MA, USA). Before each day’s ion measurement, the electrode was calibrated with five standard fluoride solutions containing 0.01, 0.10, 1, 10 and 100 ppm, respectively. After polymer films were soaked in deionized water for 24 h, 1.0 mL of each soaking solution was pipetted from 5.0 mL into a clean plastic bottle. Then, 0.1 mL total ionic strength adjustment buffer III (Thermo Orion, Beverly, MA, USA) was added to the solution and gently stirred for 5 min, and then the measurement of fluoride ions was performed [25]. The measurement was repeated three times, and the mean value was calculated. After the measurement period, the remaining soaking solution of each specimen was discarded, and the specimens were transferred to fresh deionized water (5.0 mL). The new deionized water was incubated at 37 °C for the next fluoride-release measurement. The procedure was repeated until the investigation time was reached.

#### 2.3.2. Fluoride Rechargeability

Following the 4-week period of soaking in deionized water, the specimens were collected and rinsed with deionized water, dried and individually re-immersed in 5 mL, 2 wt% NaF solution for 4 min to determine the fluoride rechargeability of specimen in each group [26]. This process stimulated the application of professional topical fluoride application, which is commonly recommended for patients with high caries susceptibility [27]. At the end of the recharging process, each specimen was thoroughly rinsed in deionized water, dried and individually immersed in 5.0 mL of deionized water and incubated at 37 °C. The fluoride content was then measured daily for 7 days. The investigation was repeated three times for each material to evaluate the consistency of the recharging abilities of the specimen. 

#### 2.3.3. In Vitro Degradation Test and Water Absorption

The degradation of the materials was assessed using weight loss measurement, while water absorption was analyzed using PBS uptake. As markers of PBS uptake and mass loss, the wet mass and dried mass of five specimens per group were measured. Prior to immersion, the initial weight (W_0_) of each specimen was measured. A 10 mL phosphate-buffered saline (PBS) solution was used as the soaking medium. The PBS volume-to-specimen mass ratio was 30 mL/g as recommended by ISO 15814 standard [28]. The specimens were kept in a PBS solution at 37 °C. For weight loss measurements, they were stored for set durations of 1, 2, 3, 4 and 8 weeks. For water absorption tests, the durations were 1 day and 1, 2, 3, 4 and 8 weeks. At the end of each investigation period, the specimens were recovered, surface-dried and reweighed (Wet mass, W_w_). After that, the specimen underwent lyophilization for 24 h and the weight was recorded (W_t_). Equations were used to calculate the percentage of water absorption (W_S_) and weight loss (W_L_):

(1) $$W_{S}\;\left(\%\right)=\frac{(W_{w}-W_{0})}{W_{0}}\;\times \;100$$

(2) $$W_{L}\;\left(\%\right)=\frac{(W_{0}-W_{d})}{W_{0}}\;\times \;100$$

where W_0_ is the initial weight of the specimen, W_w_ is the weight of wet mass at time t and W_d_ is the weight of the dried specimen [28]. The pH value of the sample solution was measured at each testing period using Thermo scientific Orion in combination with a pH meter (Orion model 4-Star benchtop pH meter, Thermo Fisher Scientific, Waltham, MA, USA).

#### 2.3.4. Surface Morphology

The scanning electron microscope (SEM, JSM-IT800, JEOL, Japan) was used to examine the surface morphology of the various blends of materials. Analysis was performed on unaged specimens and specimens that were stored in phosphate-buffered saline (PBS) at 37 °C for predetermined periods (1, 4 and 8 weeks). To achieve electro-conductivity, the samples were coated with a layer of gold prior to imaging. Analysis of surface morphology was investigated at an accelerating voltage of 15 kV.

### 2.4. Statistical Analysis

All data were analyzed using statistical software, IBM SPSS Statistics 25 (IBM corporation, New York, NY, USA). The data were subjected to a two-way ANOVA test, followed by Tukey’s HSD multiple-comparison post hoc tests. Any value of *p* < 0.05 was considered statistically significant. 

## 3. Results

### 3.1. Fluoride Release

**Figure 2:**
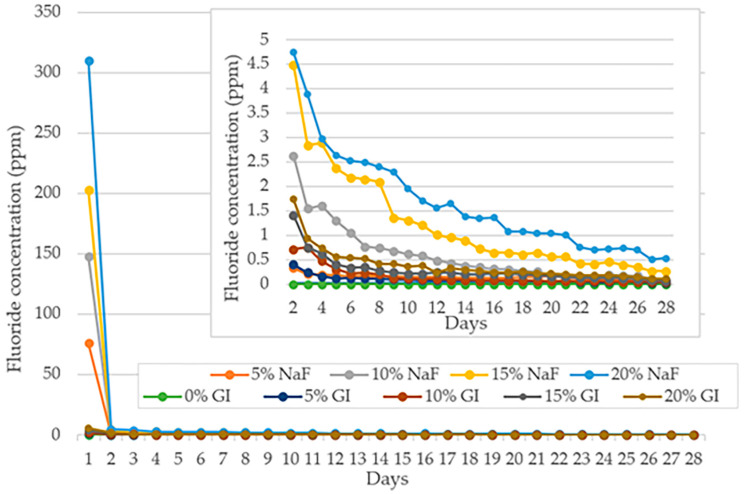
Mean fluoride concentration (ppm) over a 28-day period.

The overall trend of fluoride ion released from different test groups aged in deionized water over 28 days are presented in Figure 2. The mean ± sd of fluoride released at days 1, 7, 14, 21 and 28 are displayed in Table 1.

During the first 28 days of the experiment, all groups showed fluoride-releasing ability when compared to the control group. The highest fluoride ion concentration was noted on the first day for all groups, ranging from 76.12 ± 6.45 to 310.39 ± 34.61 ppm in PLA composite with NaF (PLA/NaF) and 1.86 ± 0.18 to 5.52 ± 0.44 ppm in PLA composite with GIC (PLA/GIC). On day 2, fluoride release decreased drastically for all experimental groups. The level of fluoride release decreased progressively over 28 days, and by the end, the level of fluoride release in all test groups had decreased to 0.53 ± 0.06 ppm or less. 

The releasing pattern of fluoride ions from PLA/NaF groups and PLA/GIC groups followed the same pattern. The pattern revealed a rapid decline in the initial phase followed by a gradual decrease in the late phase. However, it is noteworthy that the specimens in PLA/NaF groups released a significantly higher amount of fluoride ions than PLA/GIC groups and the control groups at all investigating time points (*p* < 0.05). 

Moreover, both PLA/NaF and PLA/GIC groups showed a positive correlation between fluoride releasing level and the concentration of the fluoride providing particles contained within the polymer. The highest rate of fluoride release at any time point was observed in specimens containing 20 wt% filler particles. Consequently, the release rate decreased with 15, 10, 5 and 0 wt% filler particles, respectively, as shown in Figure 2.

### 3.2. Fluoride Rechargeability

**Figure 3:**
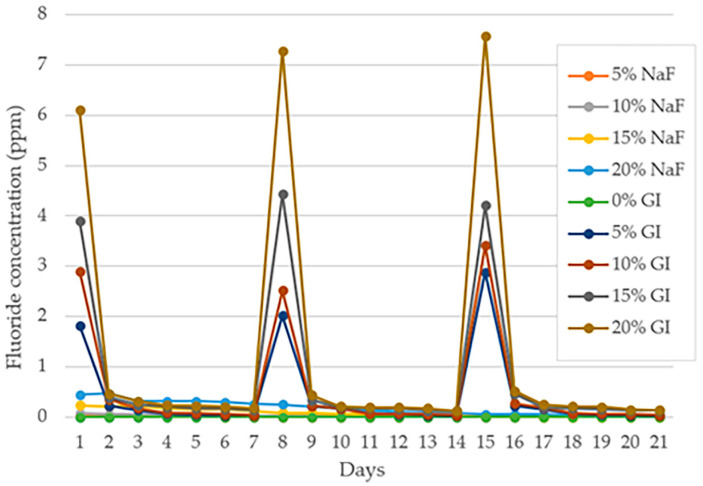
Mean fluoride concentration (ppm) after immersion in sodium fluoride for 21 days.

Figure 3 depicts the amount of fluoride ion released from specimens following the 4 min exposure to 2 wt% NaF solution for the purpose of recharging the fluoride. The procedure was repeated three times every 7 days over a period of 21 days to assess the consistency of material rechargeability. Table 2 presents the mean and standard deviation of released fluoride ion levels (ppm) on PLA/NaF, PLA/GIC and PLA (control group) following the fluoride recharging procedure at days 1, 2, 7, 8, 9, 14, 15, 16 and 21.

After exposure to 2 wt% NaF solution, the specimens of the control group showed a non-detectable level of fluoride ion in all investigating periods as presented in Table 2. 

The PLA/NaF groups did not exhibit any rechargeability. However, very low fluoride ion levels ranging from 0.02 ± 0.00 to 0.47 ± 0.09 ppm were detected in all PLA/NaF groups. The fluoride ion concentration in PLA/5%NaF, PLA/10%NaF and PLA/15%NaF composites was not detectable when measurements were taken on days 7, 14 and 21, respectively. A low level of fluoride ion with a gradually decreasing trend, from 0.44 to 0.01 ppm, was observed in PLA/20%NaF.

However, PLA/GIC groups showed rechargeability, and subsequently released fluoride ions into the soaking medium (*p* < 0.05). The amount of fluoride released after exposure to 2 wt% NaF solution was the highest on the first day, with levels of 1.82 ± 0.15, 2.90 ± 0.06, 3.90 ± 0.28 and 6.10 ± 0.37 ppm for specimens containing 5, 10, 15 and 20 wt% GIC filler, respectively. A gradual decrease in fluoride ion release was observed in all groups. 

On day 8, the specimen underwent re-exposure to 2 wt% NaF solution, the amount of fluoride released from the experimental resins significantly increased (*p* < 0.05) to 2.02 ± 0.25, 2.52 ± 0.12, 4.44 ± 0.39 and 7.28 ± 0.40 ppm for specimens with 5, 10, 15 and 20 wt% GIC filler, respectively, before decreasing significantly (*p* < 0.05) on day 9. In addition, the same trend was observed during the third exposure to 2 wt% NaF solution (day 15), where the rate of fluoride release increased substantially before decreasing the following day.

### 3.3. In Vitro Degradation Test and Water Absorption

The material degradation in the present study was evaluated by water absorption, weight loss and pH changes.

#### 3.3.1. Water Absorption

**Figure 4:**
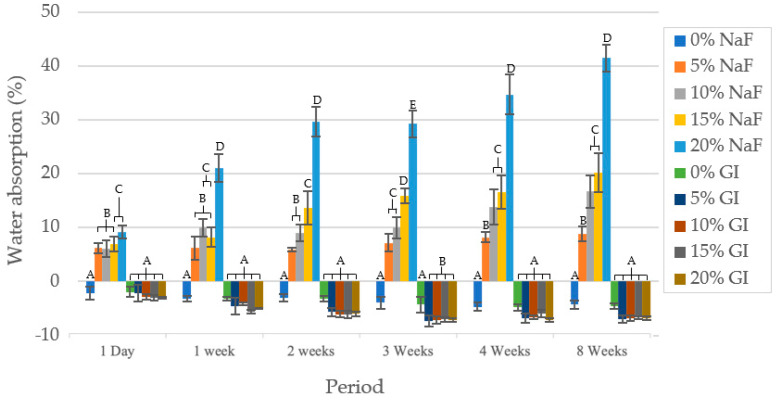
Water absorption (%) of specimen at time presented as mean values. The different letters imply statistical significance (*p* < 0.05) at the same time points.

Figure 4 shows the water absorption (%) after immersion in PBS at various time points (1 day; 1, 2, 3, 4 and 8 weeks). At each investigation time, PLA/NaF groups recorded more absorbed water than PLA/GIC groups (*p* < 0.05). On day 1, PLA/NaF groups showed a rapid increase in water absorption at 6.19 ± 0.93%, 6.14 ± 1.52%, 6.90 ± 1.39% and 9.19 ± 1.18% for 5, 10, 15 and 20 wt% NaF filler, respectively. Then, the percentage of water absorbed by all groups increased gradually over the course of each week. At 8 weeks, the water absorption of PLA/NaF containing 5, 10, 15 and 20% filler increased to 8.22 ± 1.41%, 16.66 ± 2.96%, 20.16 ± 3.58% and 34.09 ± 2.53%, respectively. The calculation of water absorption in control groups and PLA/GIC were negative. After immersion in PBS solution, the wet mass of these materials was less than the initial weight. For this reason, the water absorption cannot be calculated for these groups.

#### 3.3.2. Weight Loss

**Figure 5:**
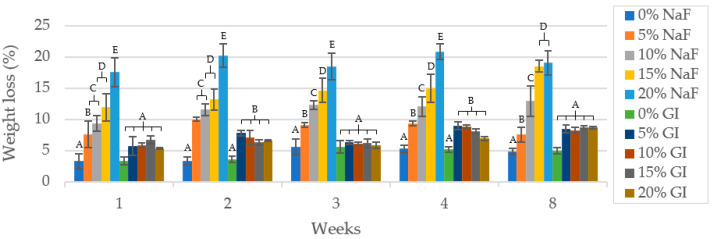
Weight loss (%) of specimen at time presented as mean values. The different letters imply statistical significance (*p* < 0.05) at the same time point.

Figure 5 depicts the weight loss (%) after immersion in PBS for up to 8 weeks. It is evident that PLA/NaF have a greater weight loss than PLA/GIC groups (*p* < 0.05). The first week of material weight loss was the most drastic, while subsequent weeks were less. At each investigation time, PLA composite with 5, 10, 15 and 20 wt% GIC filler exhibited no statistically significant differences.

#### 3.3.3. pH Change

**Figure 6:**
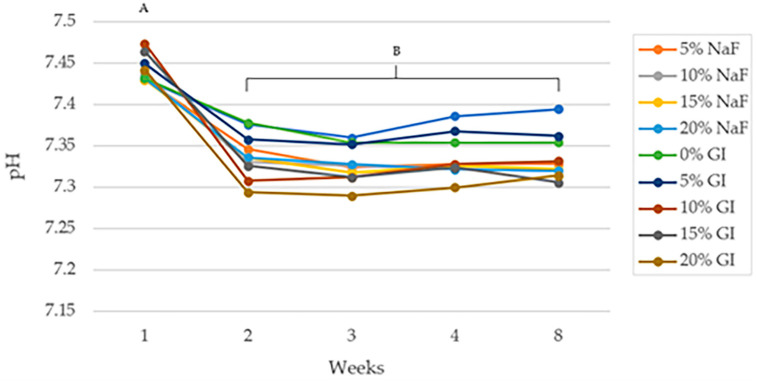
pH of specimen at time presented as mean values. The different letters imply statistical significance (*p* < 0.05).

Figure 6 shows the pH trends of the specimens after in vitro degradation in PBS for 8 weeks. During the first week, the pH slightly dropped from 7.47 to approximately 7.43 and 7.45 in PLA/NaF groups and PLA/GIC groups, respectively. The pH shift was more pronounced between the first and second weeks, with all groups dropping to 7.3. However, the pH values during weeks 2 through 8 did not statistically differ for all groups.

### 3.4. Surface Morphology

**Figure 7:**
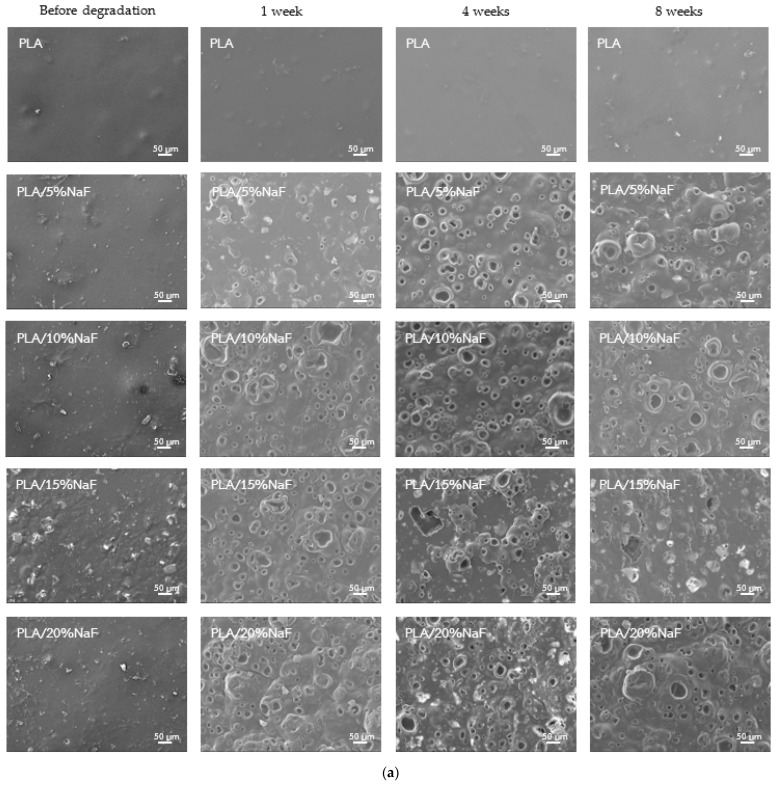

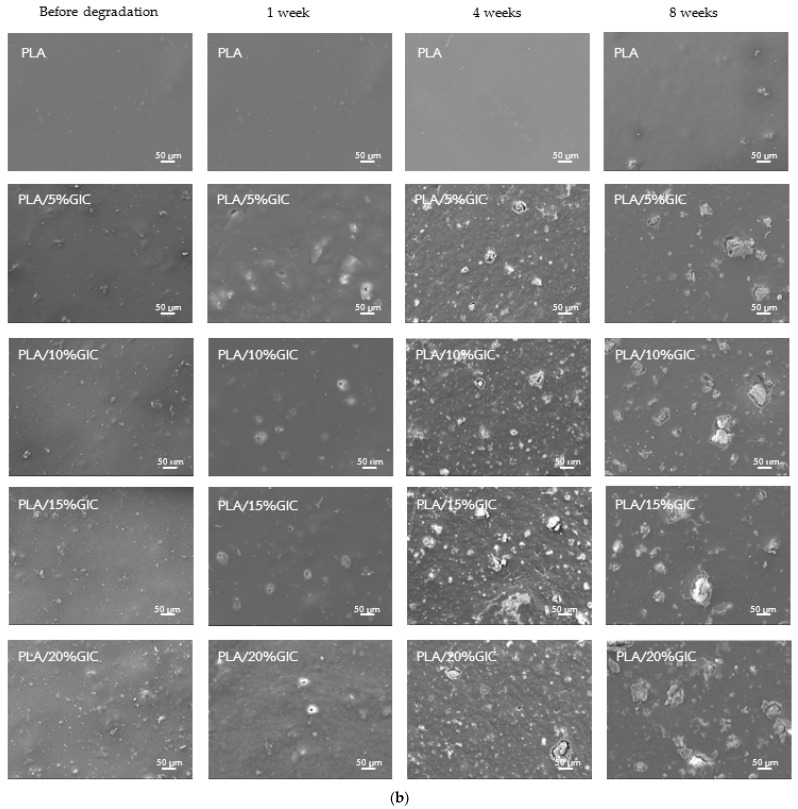
Scanning electron microscope (SEM) images of surface morphology (**a**) of PLA/NaF groups (**b**) and PLA/GIC groups.

Figure 7 presents SEM images of the surface morphology following the in vitro degradation at each investigating period. Before the degradation process, an SEM analysis of PLA (control group) showed a smooth layer morphology. In contrast, the surface of PLA/NaF and PLA/GIC specimens exhibited a roughened morphology due to the presence of fluoride, providing particles in the PLA polymer matrix. No crack lines or porous structures were observed in prepared PLA/NaF and PLA/CIG specimens. Following the immersion in PBS for an in vitro degradation test for 1 week, PLA/NaF groups exhibited spherical porous structure over the entire surface. The specimens with a lower filler content showed less porosity compared to the groups with a higher filler content. However, the porosity of PLA/15%NaF and PLA/20%NaF was not much different according to the visual evaluation. After 2 to 8 weeks of degradation, the surface morphology of PLA/NaF groups revealed comparable results to the first week. In comparison to the control group, PLA/GIC groups also displayed a more porous structure and more particles at the surface as time elapsed. However, the surfaces of the PLA/GIC groups were less porous than those of the PLA/NaF groups.

## 4. Discussion

Currently, the development of biopolymers as drug-delivery systems for medical and dental devices is gaining popularity. These biopolymer-based prosthetic devices are engineered to gradually release pharmaceutical agents or ions to the surrounding environment over an extended duration, with the primary objective of therapeutic benefits achievement [29]. Jiang et al. demonstrated this by adding clonidine hydrochloride into PLA to treat attention-deficit hyperactivity disorder (ADHD) [24]. Bakola et al. also developed a drug-eluting cardiovascular stent to reduce thrombosis. This was achieved by incorporating the anti-platelet medication of dipyridamole into a PLA scaffold [30]. In the present study, the fluoride-releasing particles, GIC and NaF, were incorporated into the PLA matrix. The material’s capability of fluoride ion release and recharge as well as the material’s in vitro degradative behavior were investigated. The primary objective of this study is to apply this developing material as the anticariogenic polymer for dental prosthesis fabrication.

Our data clearly demonstrate that all PLA/NaF and PLA/GIC composites exhibit the capability to release fluoride ions over a period of 28 days. This finding confirmed the prolonged release of fluoride ions from our developing materials. Moreover, the lowest measured level of fluoride ion in all groups (0.03 ppm) fell within the range consistent with the anti-cariogenic salivary fluoride ion concentration reported by Margolis et al. [31], which is 0.024 ppm. Other findings indicate that a sustained supply of low fluoride levels ranging from 0.03 to 0.3 mg/L for an extended length of time is most effective for caries control [9,32]. According to this, our developing PLA/NaF and PLA/GIC composites could exhibit promising potential for delivering an anti-cariogenic effect on a natural tooth structure.

The detection of fluoride ions in the soaking medium found in the present study can be attributed to the direct release from the exposed NaF and GIC particles present on the surface of the PLA polymer. In the case of PLA/NaF, the water-soluble nature of NaF particles provides an explanation for the presence of fluoride ions in the aqueous environment [33]. Conversely, the fluoride ions found in the soaking medium of the PLA/GIC composite could originate from the dispersed fluoride ions within the amorphous matrix of the set GIC particles within the PLA matrix. The infiltration and diffusion of water throughout the bulk of the set GIC particles contribute to the gradual leaching of fluoride ions into the surrounding environment [18,19,34].

The leaching out of fluoride-providing particles, particularly the water-soluble NaF salt, from the PLA polymer matrix, could be attributed to the increased material degradation observed. The formation of irregular surface structures on a micro-scale following the exposure to aqueous solution in PLA/NaF specimens results in the increasing surface roughness that enhances the entrapment of the water molecules on the material surface. Furthermore, surface defects also enhance the penetration of water molecules into the deeper layers of the polymer matrix to initiate the breakdown of the molecular structure of PLA matrix. This phenomenon known as hydrolytic degradation has been declared as the main mechanism for PLA degradation in aqueous environment [35]. 

The formation of cracks, pores or surface irregularities subsequent to exposure to an aqueous solution is indicative of surface material disintegration and subsequent material degradation [36]. Furthermore, the integration of fluoride-providing particles into the PLA polymer could potentially compromise the integrity of the polymer matrix, thereby facilitating an easier water penetration into the material [37]. This condition may provoke the hydrolytic degradation rate of the developing PLA composite compared to pure PLA.

The degradation rate of PLA depends on the water diffusion rate, PLA crystallinity, temperature, pH and its autocatalytic behavior [38,39]. In our current study, we employed various techniques to analyze the degradative behavior of the developing PLA composite, including the assessment of material weight change, alterations in surface morphology and shifts in the pH of the soaking medium following an in vitro degradation test as recommended in the previous articles [28,40,41]. Additionally, we measured the percentage of water absorption to investigate the PLA degradation rate, as a higher water absorption is assumed for increasing the attachment of water molecules on the material surface, which is referred to the higher possibility on the initiation of the hydrolytic degradation process [38].

We also monitored the change in the pH value of the soaking medium during the in vitro degradation test as an indirect method to assess the hydrolytic degradation of PLA. This value provided insights into the production of lactic acid, one of the main hydrolytic byproducts of PLA, which referred to the progression of hydrolytic degradation [42]. However, it is important to note that the present study did not reveal any significant alterations in the pH value of the soaking medium across all groups. This finding could be explained by the small degradation rate of high-molecular-weight PLA polymers as mentioned in the previous literature [38].

Our study revealed that PLA/NaF composites significantly released more fluoride ions compared to the PLA/GIC composites at all investigated time points over the 28-day period. This finding is consistent with the higher percentage of water absorption and material weight change observed on PLA/NaF composites following the in vitro degradation test. The presence of NaF, characterized as a highly soluble salt [33], is likely responsible for enhancing the composite’s water absorption through ion–dipole interactions, as discussed in earlier articles [43,44]. This higher water absorption can accelerate the hydrolytic degradation process of the PLA polymer. Additionally, the solubility of NaF may result in a more rapid release and faster depletion of the fluoride ions [12], which may indirectly contribute to the increased degradation rate [45]. Furthermore, scanning electron microscopy (SEM) analysis confirmed the formation of notable surface irregularity on PLA/NaF composites following exposure to aqueous solution. The SEM images reveal that the surface of PLA/ NaF composites exhibited greater porosity compared to the control group and GIC groups. This finding can be attributed to the leach out and detaching of the soluble NaF particles from the composite surface, thereby creating pores and irregularity in the material. These findings indicate the higher degradation behavior of PLA/NaF composites under the aqueous environment. These findings are consistent with prior research conducted by Srithongsuk et al., indicating that NaF groups were able to release more fluoride ions than GIC groups [12]. In contrast to previous studies, our findings provide new insights into fluoride release in PLA polymers rather than PMMA.

Focusing on the fluoride-releasing pattern, the similarity is observed on both PLA/NaF and PLA/GIC composites. This pattern involves an initial burst release during the first 24–48 h, followed by a gradual decline over the course of 28 days. The releasing pattern and releasing rate of PLA/GIC composites are characterized in the same way as traditional GIC [46,47,48]. This sustained release pattern of fluoride ions found in our developing composites is advantageous as it ensures a prolonged availability of fluoride ions serving tooth structure for anticariogenic action [9,32,49]. 

The degradation of the fluoride-releasing particles could play a major role in the burst release of the fluoride ions in the first week of the study, following the exposure to an aqueous environment. This hypothesis is supported by the previous articles [24,32,50]. This is in conjunction with the study from Xu et al. [51], which investigated the drug release behavior and degradation mechanism of doxorubicin loaded in a polymeric delivery system. The results showed that a burst release during the initial phase is closely related to the degradation mechanism of material compositions [51].

The dissolution of the exposed fluoride-releasing particles on the surface of the prepared specimens could be another reason for the burst release of fluoride ions at the initial stage of the experiment [50]. In PLA/NaF groups, the presence of NaF, a soluble salt, may contribute to surface dissolution and accelerate fluoride release in the initial stages. Additionally, as stated by Dionysopoulos et al. [49], amorphous GIC undergoes rapid dissolution at the surface, resulting in the accelerated release of fluoride ions. Subsequently, a more gradual release of fluoride is likely from the sustained diffusion of fluoride through the bulk cement [49]. However, it is important to note that the precise mechanism underlying the burst release of fluoride ions in the PLA polymer has not been thoroughly examined in previous studies.

By comparison, the groups with higher filler percentages of fluoride-providing particles demonstrated an increased fluoride release in both the PLA/NaF and PLA/GIC groups. These findings suggest that the amount of fluoride-providing particles in the specimen correlates with the fluoride-releasing efficiency of the developing material. The findings correspond to the study by Kamijo et al. [14], who reported the direct relationship between the filler content and fluoride ion release and recharge level. Moreover, the results of in vitro degradation support this, as they indicate that a higher filler content has a higher degradation rate. It is also important to note that fluoride leaching out of the material can potentially create voids in the matrix, and excessive release can be problematic as it can compromise the structural integrity and longevity of the material [52]. Therefore, balancing sustained fluoride release with the degradative behavior of the material is a key challenge in the development of material combined with drug-releasing particles.

Evidently, the amount of fluoride released in the PLA/GIC groups was significantly greater after the recharge regimen, whereas the control group and PLA/NaF groups showed no significant increase in fluoride release post-recharge. This is consistent with multiple studies, indicating that GIC exhibits recharging abilities [53,54,55]. Therefore, this proves that fluoride recharge occurs in the GIC filler particle. 

Our results also indicate a positive correlation between fluoride ion rechargeability and the amount of GIC particles in the PLA polymer. The findings of this investigation support Xu’s findings that materials having a higher concentration of filler content have a higher recharge capability [56]. However, the precise mechanism underlying the process of fluoride recharging in GICs is not fully understood [55,57]. It has been suggested that the recharging ability of GICs is influenced by the composition of the glass component, specifically the structure of the hydrogel layer surrounding the glass filler particles [49]. Multiple studies assume that the porosities of the GIC may exchange fluoride ions by passive diffusion, thereby indicating that materials with more pores are more permeable and can absorb more fluoride into their matrix [56,58,59]. 

The evaluation of the rechargeability across different groups exhibited a consistent pattern, demonstrating equivalent levels of fluoride ion release during each immersion in NaF, which implies that these materials have the potential to recharge fluoride ions over a sustained duration, indicating a resilient recharging ability. Given that the degradation behavior of PLA is understood to span several years, it is anticipated that this material could provide long-term utility [38]. Nevertheless, to support this assumption, longitudinal studies need to be conducted.

However, this investigation focused solely on fluoride release and did not assess the mechanical properties of the polymer composite with GIC. According to Liu et al. [60], the mechanical properties of PLA composite may be affected by the addition of filler. Therefore, future research should evaluate the material’s overall performance and durability to provide a comprehensive understanding of its suitability for dental applications. Additionally, the current study was conducted under controlled laboratory conditions and further research is needed to evaluate fluoride release in clinical settings.

## 5. Conclusions

With the limitation of this study, our developing PLA/GIC composites in all compositions provide the ability to release a sufficient level of fluoride ions to facilitate tooth remineralization theoretically. In addition, they demonstrate the potential for multiple fluoride recharging processes. Furthermore, these composites show no significant difference in terms of material morphological integrity following exposure to an in vitro degradation test in PBS for 28 days compared to pure PLA.

According to our study, the PLA composite with 20 wt% GIC particles exhibits superior fluoride ion release and rechargeability while demonstrating acceptable morphological integrity under aqueous environment. The findings from this research suggest that such a material could potentially be employed as the anticariogenic polymer for future dental applications, possibly occlusal splints or retainers.

## Figures and Tables

**Table 1:** Mean ± sd of fluoride ion concentration (ppm).

Time					
Group	1 Day	7 Days	14 Days	21 Days	28 Days
**0% NaF**	nd ^A^	nd ^A^	nd ^A^	nd ^A^	nd ^A^
**5% NaF**	76.12 ± 6.45 ^B d^	0.16 ± 0.03 ^AB c^	0.13 ± 0.02 ^AB b^	0.14 ± 0.03 ^BC bc^	0.03 ± 0.01 ^AB a^
**10% NaF**	147.66 ± 4.81 ^C e^	0.77 ± 0.16 ^D d^	0.37 ± 0.05 ^D c^	0.17 ± 0.01 ^C b^	0.09 ± 0.02 ^D a^
**15% NaF**	202.73 ± 37.61 ^D e^	2.15 ± 0.33 ^E d^	0.90 ± 0.12 ^E c^	0.56 ± 0.09 ^D b^	0.27 ± 0.05 ^E a^
**20% NaF**	310.39 ± 34.61 ^E e^	2.49 ± 0.34 ^F d^	1.39 ± 0.29 ^F c^	1.01 ± 0.18 ^E b^	0.53 ± 0.06 ^F a^
**0% GI**	nd ^A^	nd ^A^	nd ^A^	nd ^A^	nd ^A^
**5% GI**	1.86 ± 0.18 ^A e^	0.11 ± 0.02 ^AB d^	0.08 ± 0.01 ^AB c^	0.06 ± 0.01 ^AB b^	0.03 ± 0.01 ^AB a^
**10% GI**	2.19 ± 0.08 ^A e^	0.23 ± 0.04 ^AB d^	0.09 ± 0.01 ^AB c^	0.07 ± 0.01 ^AB b^	0.05 ± 0.01 ^BC a^
**15% GI**	3.75 ± 0.48 ^A e^	0.34 ± 0.03 ^BC d^	0.20 ± 0.04 ^BC c^	0.15 ± 0.02 ^BC b^	0.07 ± 0.00 ^CD a^
**20% GI**	5.52 ± 0.44 ^A e^	0.52 ± 0.06 ^C d^	0.29 ± 0.03 ^CD c^	0.20 ± 0.03 ^C b^	0.11 ± 0.02 ^D a^

nd—denotes non-detectable fluoride content. Small letters in rows indicate statistically significant differences between each day (*p* < 0.05). Big letters in columns indicate statistically significant differences between groups (*p* < 0.05).

**Table 2:** Mean values ± sd of fluoride concentration after recharge (ppm).

Time									
Group	1 Day	2 Days	7 Days	8 Days	9 Days	14 Days	15 Days	16 Days	21 Days
**0% NaF**	nd ^A^	nd ^A^	nd ^A^	nd ^A^	nd ^A^	nd ^A^	nd ^A^	nd ^A^	nd ^A^
**5% NaF**	0.04 ± 0.01 ^AB b^	0.02 ± 0.00 ^A a^	nd ^A^	nd ^A^	nd ^A^	nd ^A^	nd ^A^	nd ^A^	nd ^A^
**10% NaF**	0.07 ± 0.01 ^AB d^	0.06 ± 0.01 ^A c^	0.03 ± 0.00 ^B c^	0.02 ± 0.00 ^A b^	0.02 ± 0.00 ^A a^	nd ^A^	nd ^A^	nd ^A^	nd ^A^
**15% NaF**	0.24 ± 0.03 ^BC h^	0.21 ± 0.06 ^B h^	0.11 ± 0.01 ^C g^	0.09 ± 0.01 ^A f^	0.08 ± 0.01 ^B e^	0.03 ± 0.00 ^B d^	0.02 ± 0.01 ^A c^	0.01 ± 0.00 ^A b^	nd ^A^
**20% NaF**	0.44 ± 0.07 ^C g^	0.47 ± 0.09 ^D g^	0.26 ± 0.02 ^F f^	0.25 ± 0.03 ^A f^	0.21 ± 0.02 ^C e^	0.07 ± 0.01 ^C d^	0.06 ± 0.00 ^A c^	0.05 ± 0.00 ^A b^	0.01 ± 0.00 ^B a^
**0% GI**	nd ^A^	nd ^A^	nd ^A^	nd ^A^	nd ^A^	nd ^A^	nd ^A^	nd ^A^	nd ^A^
**5% GI**	1.82 ± 0.15 ^D d^	0.22 ± 0.04 ^B c^	0.03 ± 0.00 ^B b^	2.02 ± 0.25 ^B d^	0.22 ± 0.04 ^C c^	0.03 ± 0.00 ^B a^	2.88 ± 0.28 ^B d^	0.22 ± 0.09 ^B c^	0.02 ± 0.00 ^C a^
**10% GI**	2.90 ± 0.06 ^E g^	0.35 ± 0.04 ^C e^	0.04 ± 0.00 ^B b^	2.52 ± 0.12 ^C f^	0.22 ± 0.02 ^C c^	0.03 ± 0.00 ^B a^	3.41 ± 0.66 ^C h^	0.27 ± 0.06 ^B d^	0.03 ± 0.01 ^D ab^
**15% GI**	3.90 ± 0.28 ^F e^	0.38 ± 0.03 ^C c^	0.14 ± 0.00 ^D b^	4.44 ± 0.39 ^D f^	0.33 ± 0.06 ^D c^	0.12 ± 0.02 ^D a^	4.21 ± 0.76 ^D ef^	0.46 ± 0.04 ^C d^	0.13 ± 0.01 ^E a^
**20% GI**	6.10 ± 0.37 ^G f^	0.46 ± 0.06 ^D de^	0.17 ± 0.02 ^E c^	7.28 ± 0.40 ^E g^	0.43 ± 0.06 ^E d^	0.12 ± 0.01 ^D a^	7.58 ± 0.37 ^E g^	0.52 ± 0.05 ^C e^	0.14 ± 0.01 ^F b^

nd—denotes non-detectable fluoride content. Small letters in rows indicate statistically significant differences between each day (*p* < 0.05). Big letters in columns indicate statistically significant differences between groups (*p* < 0.05).

## Data Availability

The data presented in this study are available on request from the corresponding author.
